# Beyond Equilibrium Refractive-Index Shifts: Dynamical Information Encoded in Sensorgrams

**DOI:** 10.3390/biom16071032

**Published:** 2026-07-14

**Authors:** Giuseppina Simone

**Affiliations:** Dipartimento di Ingegneria Chimica, University of Napoli Federico II, Piazzale Tecchio 80, 80125 Napoli, Italy; giuseppina.simone@unina.it

**Keywords:** nonequilibrium plasmonic dynamics, time-resolved sensorgrams, interfacial kinetic pathways, dynamical molecular fingerprinting

## Abstract

Disordered Ag-nanowire localized surface plasmon resonance sensorgrams for glycated hemoglobin (HbA1c) detection exhibit reproducible multi-component temporal structures that cannot be fully explained within conventional equilibrium refractive-index models; in particular, the quantification of the molecular target escapes from the classical theory. The HbA1c-associated contribution emerges at earlier times while displaying slower relaxation dynamics compared with naïve hemoglobin-associated kinetics, revealing the coexistence of distinct activation and interfacial relaxation pathways. Analysis of temporal derivatives, phase-space trajectories, characteristic peak times, and relaxation times aims to suggest that the plasmonic response originates from multiple competing nonequilibrium processes evolving on different timescales. The description of the structured temporal response relies on a phenomenological framework incorporating activation and relaxation dynamics. Dynamical redistribution pathways associated with heterogeneous adsorption, hydration-shell relaxation, and plasmonic coupling within spatially non-uniform electromagnetic environments, along with resonance shift, support molecular fingerprint. The findings suggest that sensorgrams contain molecular information hidden beyond conventional equilibrium optical observables, motivating a transition toward dynamical and time-resolved approaches in plasmonic biosensing.

## 1. Introduction

Surface plasmon resonance and localized surface plasmon resonance techniques have become cornerstones of label-free biosensing [1,2,3,4]. The conventional analytical framework is elegantly simple: molecular binding at the sensor surface alters the local refractive index [2,3]. The resonance condition reduces the sensor response to a single dominant observable, such as a wavelength or angular displacement, assumed to be monotonic and directly proportional to the mass of bound analyte [5,6]. However, such an approach reduces the rich, time-dependent sensorgram to a single equilibrium readout, thereby discarding potentially valuable kinetic and conformational information encoded in the temporal response [7,8].

In a recent work on disordered Ag-nanowire localized surface plasmon resonance devices for glycated hemoglobin detection [9], the sensorgrams have exhibited reproducible multi-peak temporal structures and have induced experimental observations, basis of this perspective, but that cannot be fully explained by a static bulk refractive-index model. The measured time-dependent signals exhibited a consistent multi-peak structure, which systematically varied with the HbA1c/Hb ratio. Interestingly, the variation was especially notable with the emergence of an additional temporal component in samples containing HbA1c. Furthermore, the dominant HbA1c-associated features appeared systematically earlier in time than those observed for naive hemoglobin, suggesting that glycation of the hemoglobin influences not only the magnitude of the plasmonic response but also its temporal evolution at the sensor interface.

In this framework, the current work does not present new experimental measurements but aims to reevaluate the previously reported sensorgrams through the perspective of dynamical systems analysis [10,11,12]. The experimentally observed multi-peak responses are used as a representative case study to explore whether time-resolved plasmonic signals contain information beyond conventional equilibrium resonance shifts and to investigate the broader implications of such dynamics for plasmonic biosensing.

Overall, the quantitative analyses discussed throughout the manuscript are intended as illustrative examples supporting the broader hypothesis that goes beyond the theory of the refractive index shift.

Understanding the dynamics of the system offers valuable insights for future research directions. However, additional case studies are necessary to explore the physical origins of the observed dynamics and to assess their applicability in various plasmonic sensing models.

## 2. Limitations of Equilibrium Refractive-Index Interpretation

In classical surface plasmon resonance theory, the resonance shift is commonly described as $\Delta \lambda_{SPR}=m\Delta n(1-e^{-\frac{2d}{l_{d}}})$ where *m* is the bulk sensitivity, Δn is the refractive-index change, d  is the thickness of the adsorbed layer, and $l_{d}$ is the evanescent decay length. The formalism describes equilibrium spectral shifts associated with biomolecular adsorption. However, it does not naturally account for the emergence of multiple temporal response features or for the redistribution of signal as a function of analyte composition.

**Figure 1:**
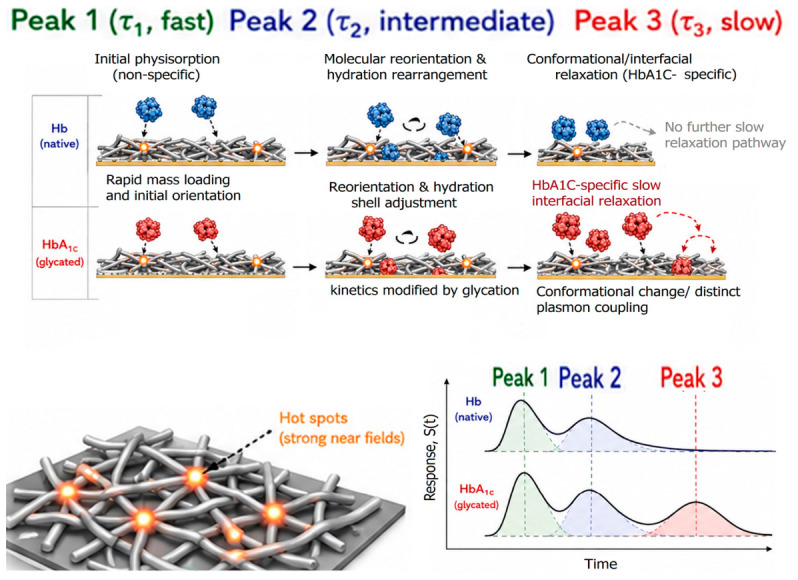
Scheme of the selective mechanism (**top**); layer with the Ag-wires (**bottom right**) and schematic of a relaxation response (**bottom left**).

In the Ag-nanowire surface, the structured design creates a highly heterogeneous electromagnetic environment, which results in localized plasmonic hotspots at the junctions of the nanowires, in nanoscale gaps, and in areas of strong near-field confinement (S1, Supporting Information) [13]. Electric field enhancement is fundamental for the generation of plasmon resonance and enables molecular measurements by influencing sensitivity, and in fact the measurements indicated that the substrate produced resonance shifts of approximately 0.37 nm for hemoglobin, while smaller, non-monotonic shifts were observed with increasing fractions of HbA1c. The sensorgrams display three main characteristics aside from the shift: (i) the emergence of three distinct peaks, (ii) the correlation between the relative sizes of these peaks and the concentration of HbA1c, rather than the overall signal amplitude, and (iii) the consistent enhancement and earlier activation of the dynamic component associated with HbA1c, in comparison to the responses of plain hemoglobin. The hypothesis is that such features are unlikely to arise from a uniform adsorption process. In fact, biomolecules interacting with different plasmonic regions of the surface with a non-uniform electromagnetic field may experience varying local field intensities, adsorption energies, and interfacial relaxation pathways, each characterized by distinct temporal signatures within the sensorgram. For samples containing both plain and glycated hemoglobin, the resulting sensorgrams exhibit reproducible multi-component temporal features. Moreover, the observed peaks suggest the presence of multiple dynamical stages in the interaction between hemoglobin species and the functionalized plasmonic surface (Figure 1). Several interpretations, which are physically plausible, can be proposed. The slowest component, referred to as Peak 1, may be linked to diffusion-limited adsorption and nonspecific interactions occurring in accessible regions of the Ag-nanowire network. Peak 2 could represent processes such as molecular reorientation, redistribution among plasmonic hotspots, or relaxation of the hydration environment after adsorption. An additional feature, primarily observed in samples containing HbA1c (Peak 3), may be associated with a glycation-related interfacial relaxation pathway. This pathway could involve changes in charge distribution, conformational flexibility, or the dynamics of hydration in glycated hemoglobin [14]. Furthermore, because the optical response of the Ag-nanowire network is dominated by localized regions of enhanced electromagnetic field intensity, the measured sensorgram is expected to be weighted toward molecular interactions occurring within or near plasmonic active different regions of the nanowire network. The resulting sensorgram may therefore reflect a superposition of responses originating from multiple local electromagnetic and interfacial environments, but whether the weighting arises solely from enhanced optical sensitivity or is additionally influenced by field-assisted molecular accumulation remains an open question requiring further investigation. 

However, consideration of alternative mechanisms is essential in this context; in fact, the observed temporal structure may arise from a combination of molecular and experimental factors, including spatial variations in local electromagnetic enhancement, nonspecific adsorption processes, and the finite temporal response of the measurement system. Baseline drift and noise amplification may additionally influence specific features of the signal, particularly in derivative representations. However, several observations suggest that the multi-peak sensorgrams are not solely the result of instrumental or statistical artifacts. The temporal features are reproducible, appear consistently across samples, and evolve systematically with HbA1c concentration. Moreover, the correlated changes observed in the sensorgrams, their temporal derivatives, phase-space trajectories, characteristic times, and relative peak area distributions collectively indicate the presence of underlying composition-dependent dynamical processes [15]. Then, if the present analysis does not uniquely identify the microscopic origin of each component, the observations support the interpretation that the sensor response reflects a superposition of multiple interfacial dynamical pathways whose relative contributions vary with molecular composition.

## 3. Sensorgram as a Superposition of Dynamical Adsorption Processes

**Figure 2:**
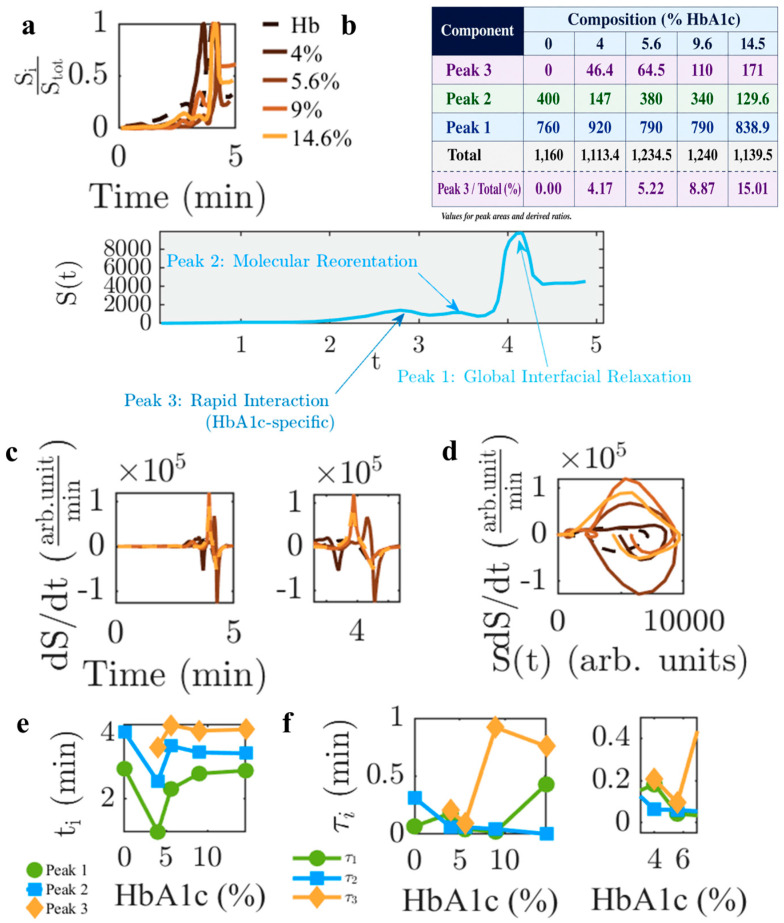
Dynamical analysis of sensorgrams for hemoglobin and glycated hemoglobin (HbA1c). (**a**) Normalized sensorgrams for naïve hemoglobin (Hb) and samples containing increasing HbA1c fractions (4–14.6%: all measurements were performed at a fixed total hemoglobin concentration of 1 mm, HbA1c/Hb fraction was varied). (**b**) (**Top**): Table demonstrating redistribution of relative peak areas among competing dynamical components as HbA1c concentration increases. (**Bottom**): Schematic of an experimental sensorgram for HbA1c-Hb sample with the associated peak interpretation. (**c**) Temporal derivatives $\left(dS/dt\right)$ of the sensorgrams, highlighting distinct kinetic regimes and revealing sharper dynamical transitions associated with HbA1c-containing samples. (**d**) Phase-space representation dS/dt-S(t) showing distinct trajectories for different HbA1c concentrations, consistent with composition-dependent interfacial relaxation pathways. (**e**) Characteristic peak times $t_{i}$ as a function of HbA1c fraction, showing systematic evolution of the temporal structure of the response. (**f**) Extracted relaxation times $\tau_{i}\;$ associated with the three dominant dynamical mechanisms.

To further examine the origin of the measured response, Figure 2 compares the experimental results with the predictions of a static equilibrium refractive-index model. For the sake of clarity, Figure 2a shows the experimental normalized sensorgrams for naive hemoglobin and samples with increasing HbA1c fraction (S2, Supporting Information). Although all sensorgrams display a multi-peak temporal structure, the relative contribution of the HbA1c-associated component, namely the peak 3, becomes progressively more pronounced as the HbA1c concentration increases. As the HbA1c concentration increases, the sensorgrams not only exhibit a greater overall amplitude, but their temporal responses change systematically as well. This includes variations in relative peak intensities, peak widths, and characteristic timescales. The crucial experimental findings shown in Ref. [9] are summarized in the table shown in Figure 2b. As the HbA1c fraction increases, the relative peak areas progressively shift from peaks 1 and 2 toward peak 3 (Figure 2b, bottom); however, the redistribution occurs while the total integrated signal varies only weakly, indicating that glycation primarily alters the partitioning of the response among competing dynamical channels, and this modification occurs without substantially changing the overall plasmonic response amplitude. Additionally, the diagram in Figure 2b (bottom) associates the kinetic phases with the sensorgram. Starting with these results, the subsequent analysis was developed. It is worth noting that while the experimental measurements were reproduced at least three times, no formal statistical methods (such as replicate-based error propagation or confidence intervals) were applied to the derived dynamical parameters.

Fundamental observations derive from the analysis of the temporal derivatives of the signal (Figure 2c). The trend of dS/dt, indeed, provides insight into the kinetics of the interfacial processes by highlighting how rapidly the signal evolves in time. HbA1c-containing samples exhibit sharper and more localized derivative extrema compared with naive hemoglobin, indicating faster temporal changes in specific stages of the adsorption/relaxation process. Emergence of the additional HbA1c-associated component produces a distinct fast dynamical contribution to the sensorgram (Figure 2c right). Additional insight is obtained from Figure 2d, where the temporal derivative dS/dt is plotted as a function of the instantaneous signal S(t). In this representation, each sensorgram traces a characteristic dynamical trajectory describing how the system evolves through adsorption and relaxation states during the sensing process. Beyond providing a qualitative visualization of dynamics, the phase-space trajectory may encode information about the underlying interfacial processes. Since the trajectory relates the sensor response to its instantaneous rate of change, its geometry reflects how rapidly the system evolves through different dynamical states. The overall extent of the trajectory along the dS/dt axis is associated with the characteristic rates of signal evolution, whereas deviations from compact or symmetric loop structures may reflect the coexistence of multiple relaxation pathways and increasing dynamical heterogeneity at the interface. Naïve hemoglobin exhibits comparatively compact and weakly distorted trajectories, consistent with a more homogeneous dynamical evolution. In contrast, samples containing increasing HbA1c fractions display progressively expanded and asymmetric loops extending toward larger positive and negative dS/dt values. Such deformation suggests that glycation influences both the rate and distribution of interfacial relaxation processes, leading to increasingly heterogeneous nonequilibrium behavior. The loop area may further reflect the degree of separation between activation and relaxation stages of the response, while trajectory asymmetry may indicate that adsorption and relaxation proceed through distinct dynamical pathways. Hence, although the present study does not attempt a quantitative analysis of these geometric characteristics, the systematic evolution of the trajectories with HbA1c concentration suggests that descriptors such as loop area, asymmetry, trajectory width, or curvature may provide experimentally useful observables for characterizing molecular interactions beyond conventional equilibrium resonance shifts. The systematic separation of the trajectories further supports the interpretation that the sensorgram encodes composition-dependent dynamical pathways rather than a single equilibrium optical response.

To further analyze the molecular response, the characteristic times provide deeper insight into the dynamics of the mechanisms involved in deposition and signal generation for the different samples.

A quantitative decomposition of the temporal response is summarized in Figure 2e,f. The temporal analysis provides insight into when the different interfacial dynamical processes become dominant during the evolution of the plasmonic response (Figure 2e). The characteristic peak times $t_{i}$, extracted from the sensorgrams as a function of HbA1c concentration, evolve systematically with increasing glycation fraction. Compared with the naïve Hb sample, the characteristic peak time decreases markedly upon the introduction of glycated hemoglobin, indicating that glycation modifies the thermodynamic balance at the nanoparticle interface and accelerates the establishment of the plasmonic response. However, further increases in HbA1c concentration produce only minor variations in the peak time, suggesting that the system rapidly reaches a regime in which the dominant interfacial process is largely insensitive to the glycation fraction. Therefore, after the initial variation, the characteristic timescale then remains approximately constant over the investigated concentration range, consistent with the establishment of a distinct interfacial regime.

The extracted relaxation times $\tau_{i}$ (Figure 2f) support the interpretation of the sensorgram as a superposition of multiple interfacial dynamical processes. The experimentally determined relaxation hierarchy, $\tau_{3}>\tau_{1}\approx \tau_{2}$, indicates that the Peak3 is described by the slowest relaxation process in the system, whereas the first two peaks exhibit comparable and substantially faster relaxation timescales. Such characteristic relaxation times describe the intrinsic decay dynamics of the individual interfacial processes and should therefore be distinguished from the temporal peak positions $t_{i}$, which indicate when the different kinetics emerge during the overall sensorgram evolution. The increasing contribution of the third relaxation channel with HbA1c concentration suggests that glycation activates an additional slow interfacial relaxation process, potentially associated with altered hydration-shell organization, modified charge redistribution, or slower conformational/dielectric equilibration of glycated hemoglobin at plasmonic hotspots. According to this interpretation, the relaxation arises from the emergence of a distinct slow dynamical pathway in the heterogeneous plasmonic environment. On this basis, the sensor response can be represented phenomenologically to capture the dominant dynamical features of the experimental sensorgrams, including their multi-peak temporal structure and their systematic evolution with HbA1c concentration. A phenomenological model, which can interpret the results, reads

(1) $$S(t)=S_{0}+\sum_{i}A_{i}(1-e^{-\frac{t}{\tau_{r,i}}})e^{-\frac{t}{\tau_{d,i}}}$$

where $A_{i}$ denotes the amplitude of the i-th dynamical component, $\tau_{r,i}$ its characteristic rise time, and $\tau_{d,i}$ its characteristic decay (relaxation) time (S3, Supporting Information). Each term therefore represents a distinct interfacial process contributing to the overall sensor response, characterized by an initial signal buildup followed by relaxation on a characteristic timescale. The principal aim of model is to compare relative trends across Hb/HbA1c compositions more than to extract unique microscopic rate constants. Accordingly, the parameters should be interpreted as effective descriptors of interfacial dynamics rather than mechanistically unique states.

In the model of Equation (1), the rise times describe the activation or buildup dynamics of the individual interfacial contributions, whereas the decay times characterize their subsequent relaxation toward equilibrium. Physically, such contributions may originate from coupled processes occurring on different timescales, including diffusion toward the surface, adsorption onto localized plasmonic hotspots, molecular reorientation after binding, hydration-shell rearrangement, and local dielectric relaxation within the interfacial layer. Because the Ag-nanowire ensemble generates highly heterogeneous electromagnetic confinement, biomolecules interacting with different regions of the substrate may experience distinct adsorption energies and relaxation pathways, naturally producing the observed structured temporal response. Hence, the structured temporal features cannot be readily explained within the conventional equilibrium refractive-index framework, which effectively treats the adsorbed biomolecular layer as a homogeneous dielectric slab characterized by a single average optical response. Overall, while this approximation is effective in reproducing the overall resonance shift, it does not capture (i) the emergence of multiple peaks in the sensorgram, (ii) the redistribution of peak amplitudes under an approximately conserved total integrated response, or (iii) the presence of distinct relaxation times associated with different dynamical contributions. Beyond the identification of individual kinetic events, the present analysis provides a broader physical interpretation of HbA1c quantification in plasmonic sensorgrams. As shown in Figure 2, although the total integrated plasmonic response remains approximately constant across samples, the relative contributions of the individual temporal components exhibit a systematic redistribution with increasing HbA1c fraction. The glycation-associated component shows a monotonic increase with HbA1c concentration ($A_{3}\propto C_{HbA1c}$), accompanied by a corresponding reduction in the relative weight of the other components, as the empirical observation of the Hb/HbA1c system has displayed. Nevertheless, the approximate conservation of the total integrated response suggests that glycation primarily redistributes the signal among competing interfacial dynamical pathways without significantly altering the overall optical response. Based on these observations, increasing HbA1c concentration effectively shifts the occupancy of interfacial dynamical states, resulting in a transfer of spectral weight toward the glycation-associated component while preserving the global response. Whether the redistribution reflects a general feature of heterogeneous plasmonic interfaces or is specific to the present system remains an open question for future work.

The redistribution hypothesis is independently supported by the temporal and kinetic analyses summarized in Figure 2e,f. The characteristic peak times $t_{i}$ evolve systematically with increasing HbA1c concentration, revealing that the different kinetic regimes progressively shift within the temporal structure of the sensorgram as glycation increases. In parallel, the extracted relaxation hierarchy, $\tau_{3}>\;\tau_{1}\approx \;\tau_{2}$ suggests that the glycation-associated contribution corresponds to a slower relaxation pathway compared with the faster naïve hemoglobin-associated channels. The coexistence of earlier temporal emergence together with longer relaxation dynamics suggests that the HbA1c-associated process is not simply an independent additive signal, but rather a distinct interfacial pathway characterized by delayed equilibration and persistent dielectric/interfacial relaxation. As the HbA1c concentration increases, a progressively larger fraction of the total plasmonic response evolves through this slower dynamical channel, naturally producing the experimentally observed redistribution of peak areas.

In the temporal domain, the different events of the sensorgram may therefore be interpreted as competing adsorption and relaxation pathways whose relative occupation probabilities depend on molecular composition. Increasing HbA1c concentration does not simply produce a larger equilibrium optical perturbation; instead, it progressively shifts the interfacial system toward dynamical configurations associated with the glycation-induced relaxation pathway. Such behavior is physically consistent with heterogeneous electromagnetic field generated by the Ag-nanowire network, including localized plasmonic hotspots, which, then, is expected to modulate the relative occupation of the competing interfacial dynamical pathways. Such substrates can favor specific interfacial dynamical states over others. Therefore, glycation-induced changes in hemoglobin, encompassing altered electrostatic properties, hydration-shell organization, and conformational dynamics, may modify its interaction with heterogeneous plasmonic regions.

In this perspective, the relative peak areas become effective in describing the probability that the interfacial system evolves through a particular adsorption/relaxation pathway during the sensing process. The HbA1c percentage is therefore encoded not in the absolute magnitude of a single equilibrium optical observable, but rather in the evolving balance among competing dynamical channels within the temporal structure of the sensorgram.

More broadly, the observations suggest that resonance biosensing may access molecular information that is not captured by conventional equilibrium refractive-index analysis, motivating a shift toward a dynamical interpretation of plasmonic–molecular interactions. An important open question is which measurable molecular properties ultimately govern the observed dynamical signatures. The present analysis shows that different analyte compositions generate distinct sensorgram morphologies, relaxation times, and phase-space trajectories; however, the specific physicochemical descriptors underlying these features remain to be identified. Several molecular properties may contribute to this behavior, including adsorption and free energy, surface charge distribution, hydration-shell organization, dielectric relaxation, conformational flexibility, molecular compressibility, and interfacial binding kinetics. Such factors collectively determine how biomolecules interact with heterogeneous plasmonic environments and may therefore shape the characteristic timescales, amplitude distribution, and temporal evolution of the sensorgram.

A key future direction is the development of quantitative frameworks linking molecular properties to experimentally accessible sensorgram features. In this context, recent work by Wang et al. [16] demonstrated that kinetic observables associated with membrane interfacial processes can be related to thermodynamic quantities, providing a predictive framework for complex interfacial dynamics. Although the physical system differs from plasmonic biosensing, the underlying conceptual approach is directly relevant. An analogous framework for plasmonic resonance sensing would aim to connect molecular thermodynamic, structural, mechanical, or electrostatic properties to dynamical observables such as peak positions, relaxation times, relative component contributions, and phase-space descriptors. Establishing such relationships would extend plasmonic sensing beyond qualitative dynamical fingerprinting toward a predictive methodology in which molecular properties can be inferred from the temporal structure of the sensor response. In this perspective, the objective is not only to identify dynamical signatures, but also to uncover the physical principles governing their emergence and to translate them into quantitative descriptors of molecular behavior at complex interfaces.

## Data Availability

All data produced in this research have been included in this manuscript.

## Supplementary Materials

The following supporting information can be downloaded at: https://www.mdpi.com/article/10.3390/biom16071032/s1, Section S1: The experimental setup and the measurements; Figure S1. Setup and experiments; Section S2. Data Analysis; Section S3. Relaxation-Time Estimation.

## References

[1] Simone G. (2024). Trends of Biosensing: Plasmonics through Miniaturization and Quantum Sensing. Crit. Rev. Anal. Chem..

[2] Anker J.N., Hall W.P., Lyandres O., Shah N.C., Zhao J., Van Duyne R.P. (2008). Biosensing with Plasmonic Nanosensors. Nat. Mater..

[3] van de Donk O., Zhang X., Simone G. (2019). Superstructure of Silver Crystals in a Caged Framework for Plasmonic Inverse Sensing. Biosens. Bioelectron..

[4] Haes A.J., Hall W.P., Chang L., Klein W.L., Van Duyne R.P. (2004). A Localized Surface Plasmon Resonance Biosensor: First Steps toward an Assay for Alzheimer’s Disease. Nano Lett..

[5] Homola J. (2008). Surface Plasmon Resonance Sensors for Detection of Chemical and Biological Species. Chem Rev..

[6] Mayer K.M., Hafner J.H. (2011). Localized Surface Plasmon Resonance Sensors. Chem. Rev..

[7] Mcoyi M.P., Mpofu K.T., Sekhwama M., Mthunzi-Kufa P. (2025). Developments in Localized Surface Plasmon Resonance. Plasmonics.

[8] Yu X., Yuan Y., Zhou J., Chang M., Zeng S., Zhuang S. (2026). Sensitivity Enhancement of Surface Plasmon Resonance Biosensors Based on Versatile Nanostructures: Principle, Fabrication, and Illustrative Applications. Microsyst. Nanoeng..

[9] Zhang H., Li D., Yang Y., Chang H., Simone G. (2020). On-Resonance Islands of Ag-Nanowires Sense the Level of Glycated Hemoglobin for Diabetes Diagnosis. Sens. Actuators B Chem..

[10] Gassner C., Karlsson R., Lipsmeier F., Moelleken J. (2018). Beyond Conventional Dose-Response Curves: Sensorgram Comparison in SPR Allows Single Concentration Activity and Similarity Assessment. J. Pharm. Biomed. Anal..

[11] Shi X., Kuai L., Xu D., Qiao Y., Chen Y., Di B., Xu P. (2025). Surface Plasmon Resonance (SPR) for the Binding Kinetics Analysis of Synthetic Cannabinoids: Advancing CB1 Receptor Interaction Studies. Int. J. Mol. Sci..

[12] Simone G. (2021). Surface Plasmon Resonance Study for a Reliable Determination of the Affinity Constant of Multivalent Grafted Beads. Soft Matter.

[13] Simone G., de Ruijter P. (2020). Plasmon Resonance Excitation Enhances Raman Emission and Amplifies the Molecular Vibration on Au (111) Film. Appl. Surf. Sci..

[14] Hojjat Jodaylami M., Masson J.-F., Badia A. (2025). Surface Plasmon Resonance Sensing. Nat. Rev. Methods Prim..

[15] Abdalla S., Farsaci F., Tellone E., Shirbeeny W., Hassan A.M., Bahabri F., Kandil S. (2022). Hemoglobin Glycation Increases the Electric Charges on Red Blood Cells: Effects of Dielectric Polarization. Mater. Chem. Phys..

[16] Wang K., Villanueva M.E., Caporaletti F., White R.P., Lipson J.E.G., Napolitano S., Losada-Pérez P. (2026). Glassy Dynamics as a Predictive Framework for Lipid Exchange Across Membranes. Small.

