# Supplementary material for: Beyond Equilibrium Refractive-Index Shifts: Dynamical Information Encoded in Sensorgrams
Source: Biomolecules. 2026 Jul 14;16(7):1032. doi: 10.3390/biom16071032 (PMC13406583; doi:10.3390/biom16071032)
Supplement: Supplementary file 1 [file biomolecules-16-01032-s001.zip › biomolecules-4355359-supplementary.pdf]

biomolecules

Commentary

Beyond Equilibrium Refractive-Index Shifts: Dynamical Information Encoded in Sensorgrams

Giuseppina Simone

S1. The experimental setup and the measurements

The analysis presented in this work is based on results previously published in Reference 7, where readers can find a detailed description of the fabrication and characterization of the experimental platform, as well as the plasmonic investigation. For clarity, a summary of the experiment is shown in Figure S1a, which displays a schematic diagram of the experimental setup, including the chip itself. This diagram illustrates how the samples are placed on the plasmonic chip. Figure S1b presents a model of hotspots.

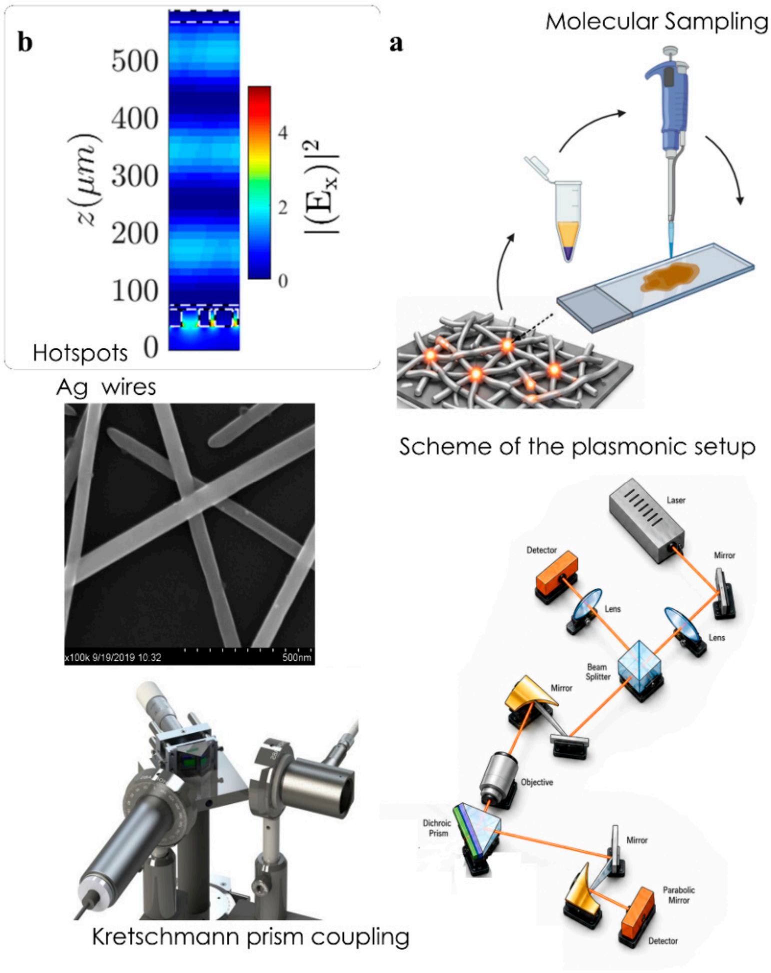

Figure S1. Setup and experiments. a. Setup with details on the Kretschmann prism coupling and sensor assembling. Manual sampling is also illustrated. b. Hotspots in proximity of the wire-to-wire gap.

It is worth noting that the experimental setup does not include temperature control. In fact, the temperature was not monitored during the present experiments. Nevertheless, the measurements were performed using a Class 2 continuous-wave laser with a maxi-

mum optical power of 1 mW. An upper-bound estimate indicates that complete conversion of the incident optical power into heat over a 5 min acquisition would correspond to a temperature increase of less than approximately 1 °C for a typical aqueous sample volume, even under the unrealistic assumption of perfect absorption and negligible heat dissipation. Therefore, significant bulk heating is unlikely to represent the dominant origin of the observed sensorgram dynamics.

S2. Data Analysis

All signal processing and kinetic analyses were performed in MATLAB (R2025, MathWorks) using a consistent workflow applied to all hemoglobin (Hb) and glycated hemoglobin (HbA1c) sensorgrams. To reduce high-frequency noise while preserving the underlying temporal structure of the sensorgrams, a Savitzky–Golay smoothing filter was applied, with a fixed window length of nine data points. The procedure was applied uniformly across all datasets and was chosen because it preserves peak shape and local extrema more effectively than simple moving-average filtering. No additional baseline subtraction or detrending was performed beyond this smoothing step. For comparative visualization, each smoothed sensorgram was subsequently normalized by its maximum value, such that the normalized signal was defined as $S_{norm} = \frac{S_i(t)}{S_{max}(t)}$. This normalization was applied independently to each sample to enable direct comparison of temporal features without influencing the relative timing or morphology of the sensorgram structures. Temporal derivatives were computed from the smoothed signals using a finite-difference approximation implemented via gradient function, which evaluates the numerical derivative as a central difference estimate of ΔS/Δt. The resulting derivative curves were used both for kinetic visualization and for constructing phase-space representations in which dS/dt was plotted as a function of the instantaneous signal S(t), thereby providing a two-dimensional dynamical description of the sensor response. Therefore, the number of temporal features, three peaks, is not a fitted parameter of a kinetic model but a reproducible structural property of the processed sensorgrams, identified directly from the consistently observed peak structure in the experimental data. No global fitting procedure or parametric peak decomposition was performed. The procedure has been clarified better in the Supporting Information. Peak identification was performed using an interactive manual selection procedure implemented. This approach was adopted to ensure consistent identification of reproducible multicomponent structures across samples exhibiting different signal morphologies. For hemoglobin (0% HbA1c), two dominant peaks were selected, whereas for all HbA1c-containing samples (4%, 5.6%, 9%, and 14.6%), three peaks were identified in accordance with the observed multi-component structure of the sensorgrams. Peak positions were recorded as the time coordinates of the selected points, while peak amplitudes corresponded to the signal values at those coordinates. Although such procedure introduces ensures consistent peak attribution across heterogeneous signal profiles where automated peak detection is not robust. Peak areas were computed by numerical integration of the signal within time intervals defined by successive peak positions. Integration was performed using the trapezoidal rule, and for the final peak the integration extended from the last selected peak to the end of the recording window. Normalized peak areas were then obtained by dividing each individual peak contribution by the total integrated signal of the corresponding sensorgram, enabling comparison of relative spectral weight distributions across samples. In addition, the total sensor response was quantified as the integral of the full smoothed signal over time. Peak areas (A_i) were computed by numerical integration of the signal S(t) within time intervals defined by successive peak positions. For intermediate peaks, the peak area was calculated as $A_i = \int(t_i \rightarrow t_{i+1} S(t)dt$ where t_i denote the positions of two consecutive peaks. For the final peak, integration was extended from the last selected peak to the end of the recording window, $A_i =$

$\int(t_i \rightarrow t_{end} \; S(t)dt.$ Numerical integration was performed using the trapezoidal rule applied to the smoothed sensorgram. Relative peak contributions were then calculated as $A_{i,rel} = \frac{A_i}{\sum A_j}$, where $\sum A_j$ represents the sum of all peak areas within the corresponding sensorgram. Normalization enabled comparison of the relative spectral weight distribution among the different dynamical components across samples. In addition, the total sensor response was quantified as the integral of the full smoothed signal over the entire recording period.

S3. Relaxation-Time Estimation

Characteristic relaxation times were estimated directly from the temporal decay of the individual sensorgram peaks using a model-independent, data-driven approach. Following signal smoothing with a Savitzky–Golay filter and identification of the relevant peaks, the sensorgram was segmented according to the selected peak positions. For each peak located at time t_i, the post-peak signal segment was normalized to its local maximum value S_{peak}, yielding a dimensionless decay profile $S^*(t) = S(t)/S_{peak}$. The relaxation time associated with the i-th peak, τ_i, was defined as the elapsed time required for the normalized signal to decrease from its maximum value to the threshold 1/e (≈ 0.368), such that $\tau_i = \frac{t_1}{e} - t_i$, where $\frac{t_1}{e}$ denotes the first time point at which $S^*(t) \leq 1/e$. This threshold-based criterion provides an effective estimate of the characteristic decay timescale associated with each dynamical component identified in the sensorgram. If the normalized signal did not reach the 1/e threshold within the available observation window, the relaxation time was approximated as the duration of the accessible segment beyond the peak position, providing a lower-bound estimate of the relaxation process. Importantly, the reported relaxation times (τ_1, τ_2, and τ_3) were extracted directly from the experimental time traces and were not obtained through exponential fitting, peak-width analysis, or any other parametric kinetic model. Consequently, these values should be interpreted as effective phenomenological descriptors of the observed relaxation dynamics rather than intrinsic kinetic rate constants. The use of a uniform threshold-based procedure across all datasets ensures methodological consistency and enables robust comparison of relaxation behavior among different samples while maintaining a direct, assumption-minimized connection to the experimental measurements.
